# Enucleation and evisceration at a tertiary care hospital in a developing country

**DOI:** 10.1186/s12886-015-0108-x

**Published:** 2015-09-11

**Authors:** Osama H. Ababneh, Eman A. AboTaleb, Mohammad A. Abu Ameerh, Yacoub A. Yousef

**Affiliations:** Department of Ophthalmology, Jordan University Hospital, The University of Jordan, Amman, Jordan; Department of Ophthalmology, Sana’a University, Sana’a, Yemen; Department of Surgery, King Hussein Cancer Center, Amman, Jordan

## Abstract

**Background:**

To analyze the demographics, indications, and surgical outcomes of anophthalmic surgery (enucleation and evisceration) at Jordan University Hospital during a 5-year period.

**Methods:**

We conducted a retrospective chart review of patients who had undergone evisceration or enucleation between August 2006 and June 2011. The data collected included age at time of surgery, sex, affected eye, surgical indication, implant size, and postoperative complications.

**Results:**

Anophthalmic surgery was performed for 68 eyes of 67 patients during the study period (42 (62 %) eviscerations and 26 (38 %) enucleations). Forty-three patients (64 %) were men, and 40 (59 %) eyes were right eyes. Trauma was the leading cause for anophthalmic surgery in 40 % of cases followed by a blind painful eye secondary to glaucoma (19 %) in the enucleation group and endophthalmitis (28.6 %) in the evisceration group. The most common anophthalmic surgery complication was wound dehiscence in 11.5 % of patients in the enucleation and 9.5 % in the evisceration groups. The mean and median sizes of the implants for evisceration were 16.6 and 18.0 mm, respectively; for enucleation, both were 20 mm.

**Conclusions:**

Evisceration was the preferred anophthalmic surgery in our series unless contraindicated. Trauma was the most common predisposing factor for evisceration and enucleation in our tertiary care center followed by blind painful eyes and endophthalmitis. The most common complication was wound dehiscence in both groups.

## Background

Enucleation, the surgical removal of the entire globe, and evisceration, the complete removal of the intraocular contents through a corneal or a scleral incision, with preservation of the conjunctiva, sclera, extraocular muscles, orbital fat and the optic nerve, have been acceptable therapeutic modalities to treat various ocular conditions, such as; intraocular tumors (enucleation only), severe eye trauma, and blind, painful, cosmetically disfiguring eyes over the last two centuries [1, 2].

Evisceration is a technically easier surgery to perform compared with enucleation, causes less disruption of the orbital anatomy, and may have fewer complications such as ptosis, implant migration and extrusion, socket contracture, and the deep superior sulcus syndrome [3, 4]. The current practice patterns that reflect the recent literature and historic trends were revealed by a recent national survey of evisceration and enucleation practice patterns in the United States, where two-thirds of ophthalmic plastic and reconstructive surgeons preferred evisceration over enucleation when the underlying cause of painful eye was benign; and implant exposure was the most commonly encountered complication after both surgeries [5, 6].

The usual indications for evisceration are unresponsive endophthalmitis and for improvement of cosmesis in a blind eye, while enucleation is indicated for the previous two conditions as well as for painful blind eye, intraocular malignancy, severe ocular trauma, phthisis with degeneration, and in congenital anophthalmia or severe microphthalmia [1, 2, 4–7]. In a review of 24,444 enucleation cases over a 55-year period, Spraul and Grossniklaus [7] found trauma to account for 40.9 % of cases, whereas tumors were the cause of enucleation in 24.2 % of cases. In the other hand Chaudhry et al. [8] found endophthalmitis to account for 45.5 % of cases, whereas phthisis bulbi and trauma together were the cause of 39.5 % of cases.

We conducted this retrospective study to determine the underlying ocular conditions leading to anophthalmic surgery (enucleation and evisceration) in a tertiary care center in Jordan and to evaluate the surgical outcomes of both surgeries.

## Methods

A retrospective review of the medical records of 67 patients who had undergone anophthalmic surgery (evisceration or enucleation) between August 2006 and June 2011 in the Department of Ophthalmology, Jordan University Hospital was carried on. All patients who had their anophthalmic surgery at Jordan University Hospital during the study period were included. The study protocol adhered to the tenets of the Declaration of Helsinki and was approved by the local institutional review board and ethics committee (IRB at Jordan University Hospital and The Faculty of Medicine). Patients were only excluded if they failed to complete 12 months of follow up. Patients who had a second anophthalmic procedure (in cases of implant exchange); the first procedure was used for statistical analysis. The data collected included the patients’ demographics, indications for anophthalmic surgery, type of anophthalmic surgery, affected eye, size and type of orbital implant, duration of follow-up, and complications encountered during the follow-up period. Enucleation was performed mainly for patients for whom evisceration was contraindicated; when an intraocular tumor could not be ruled out by clinical examination or imaging, or when it was difficult to perform evisceration due to severe phthisis bulbi or irreparable ocular rupture. When performing a fundus examination to rule out an intraocular tumor was impossible due to media opacity, a B-scan ultrasonography and orbital magnetic resonance imaging (MRI) were performed preoperatively. Statistical analysis was performed using the IBM SPSS Statistics version 20.0 (IBM Corporation, *Somers*, New York, USA) and the independent samples *t*-test was used to compare the enucleation and evisceration statistical means and fisher’s exact test to compare postoperative anophthalmic surgical complications.

**Table 1:** Patients’ demographic data

Characteristic	Enucleation (26 eyes)	Evisceration (42 eyes)
Mean age (years)	25.24 ± 16.50	47.12 ± 24.11
Range (years)	1–63	3–85
Men/women	15/10	28/14
Affected eye	14 right/12 left	26 right/16 left

## Surgical technique

All patients provided fully informed written consent for surgery including an explanation of the possible postoperative complications. All surgeries were performed under general anesthesia.

For enucleation, a retrobulbar injection of 5 ml of 50:50 Bupivacaine HCL with lidocaine and 1:100,000 adrenaline were administered, a 360° peritomy was performed at the limbus and the four quadrants were bluntly dissected to release the conjunctiva and Tenon’s capsule from the globe. The medial and lateral recti muscles were identified and sutured near the insertion with 4–0 silk suture. Bipolar cautery was applied to the four recti muscles near their insertions where they were cut and disinserted from the globe. The dissection continued posteriorly, and the superior and inferior oblique muscles were cut. The globe was retracted anteriorly using the two silk sutures and a long curved hemostat was secured around the optic nerve as posterior as possible. The optic nerve was transected with a blunt curved enucleation scissor while the medial and lateral recti insertions were held under traction using the silk sutures and the loose globe was removed. After hemostasis was achieved with bipolar diathermy and pressure applications with icepacks, a sterile, silicone implant was inserted primarily in the orbital socket posterior to the posterior Tenon’s capsule to replace the lost orbital volume. The implant size was estimated depending on the age of the patient and the size of the orbit using the trial set that allows tension-free closure of the anterior surface tissues. Tenon’s capsule (posterior and anterior) and conjunctiva were closed in layers using 5–0 and 6–0 polyglactin sutures (Vicryl, Ethicon Inc.), respectively.

For evisceration, a 360° peritomy was created, and a stab incision was made in the sclera about 1 to 2 mm from the surgical limbus with a no. 11 blade scalpel. The incision was continued circumferentially around the limbus with Wescott scissors. An evisceration spoon was used to separate the uveal tissue from the scleral shell, and the globe contents were removed. The optic disc was cauterized and the inside of the scleral shell was cleaned and debrided. Anterior relaxing incisions were made in the sclera nasally and temporally, avoiding the medial and lateral rectus muscles. An appropriately sized silicone sphere implant that allowed scleral closure without undue tension was inserted primarily (as with enucleation) in All cases. The scleral shell was closed with 5–0 polyglactin (Vicryl, Ethicon Inc.) in a horizontal mattress sutures. The anterior Tenon’s capsule and conjunctiva were closed in layers with 5–0 and 6–0 polyglactin (Vicryl, Ethicon Inc.) sutures, respectively. The entire globe (enucleation) or intraocular contents (evisceration) were sent for histopathological examination in all cases.

A medium or large conformer was inserted, and antibiotic ointment was placed on the ocular surface. Two frost sutures (4–0 silk) were applied over a bolster for 2 weeks and an eye patch was applied for 1 week. Intraoperatively, the patients received an intravenous broad-spectrum antibiotic and were discharged on oral antibiotics for 10 days. The conformer was maintained for 6 to 8 weeks. Further follow-up visits were scheduled for 1, 2, 4, 6, and 8 weeks, 3 months, and every 3 months thereafter to examine the socket for possible complications.

## Results

Sixty-eight eyes of 67 patients underwent enucleation or evisceration at Jordan University Hospital between August 2006 and June 2011. The mean follow-up duration was 24 months (range, 12–42 months). Twenty-five patients (26 eyes, 38 %) underwent enucleation and 42 patients (42 eyes, 62 %) underwent evisceration. The patients’ demographics are summarized in Table 1. In the enucleation group, one woman had bilateral enucleation secondary to severe facial trauma while in motor vehicle accident. The mean age at the time of surgery was 38.8 ± 24.8 years. (Range, 1–85 years). The difference in mean age between the evisceration (47.12 ± 24.11 years) and enucleation (25.24 ± 16.50 years) subgroups was statistically significant (*p* = 0.001). Two-thirds of our patients (69 % of eviscerated eyes and 64 % of enucleated eyes) had a history of a previous ocular surgery mainly; ruptured globe repair, cataract surgery, or penetrating keratoplasty.

**Table 2:** Indications for enucleation and evisceration

Indication	Evisceration/enucleation No. of eye	Evisceration/enucleation %
Severe trauma	14/13	33.3 % / 50 %
Glaucoma	5/5	12 % / 19.2 %
Endophthalmitis	12/1	28.6 % / 3.8 %
Keratitis	10/2	23.8 % / 7.7 %
Behcet’s disease	1/2	2.4 % / 7.7 %
Tumors	0/3	0 % / 11.5 %
Total	42/26	100 % / 99.9 %

Severe trauma was the leading reason for anophthalmic surgery at our hospital in 40 % (*n* = 27/68) of cases; (50 % (*n* = 13/26) in the enucleation group and 33.3 % (*n* = 14/42) in the evisceration group). The second most common indication for enucleation was a blind, painful, irritable eye with phthisis bulbi and degeneration secondary to absolute glaucoma and accounted for 19 % (5/26) of cases. In the evisceration group, endophthalmitis was the second most common indication in 28.6 % (12/42) followed by keratitis (23.8 %) (Table 2). Two of the cases of enucleation had intraocular retinoblastoma and one had a choroidal melanoma. The indications of anophthalmic surgery (enucleation or evisceration) according to age and gender are shown in Tables 3 and 4, respectively.

**Table 3:** Indications for Enucleation and Evisceration surgery according to age

Age (Years)	Trauma	Endophthalmitis	Glaucoma	Keratitis	Behcet’s disease	Tumors	Total
1–9	1	1	1	2	0	2	7
10–19	8	1	0	1	0	0	10
20–29	6	1	2	0	0	0	9
30–39	6	0	2	1	2	0	11
40–49	2	1	3	1	0	1	8
50–59	0	1	1	3	0	0	5
60–69	1	3	1	0	1	0	6
70–79	2	4	0	4	0	0	10
80–89	1	1	0	0	0	0	2
Total	27	13	10	12	3	3	68

**Table 4:** Indications for Enucleation and Evisceration surgery according to sex

Surgery	Trauma	Endophthalmitis	Glaucoma	Keratitis	Behcet’s disease	Tumor	Total
Evisceration							
Men	10	5	4	7	1	0	27
Women	4	7	1	3	0	0	15
Enucleation							
Men	6	1	3	0	2	3	15
Women	7	0	2	2	0	0	11
Total	27	13	10	12	3	3	68

**Table 5:** Postoperative anophthalmic surgical complications

Complication	Evisceration Incidence/%	Enucleation Incidence/%	*P* value (Fisher’s exact test)
No complication	30/71.4	16/61.5	0.43
Wound dehiscence	4/9.5	3/11.5	1.00
Implant exposure	2/4.8	1/3.8	1.00
Implant extrusion	1/2.4	2/7.7	0.55
Implant migration	0/0	1/3.8	0.38
Infection	2/4.8	1/3.8	1.00
Deep superior sulcus	3/7.1	2/7.7	1.00
Total	42/100	26/100	

**Fig. 1:**
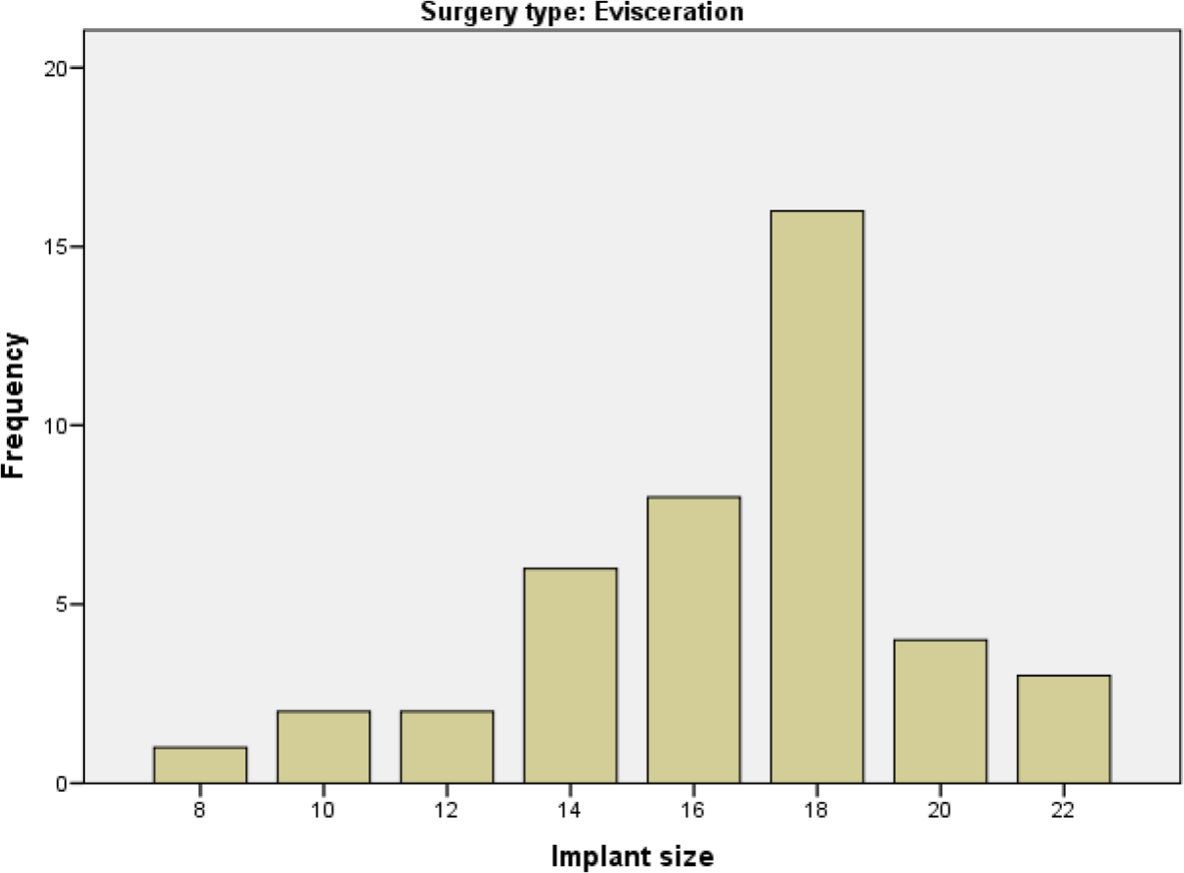
The sizes of the silicone implant spheres in millimeters and their frequency in the evisceration subgroup

**Fig. 2:**
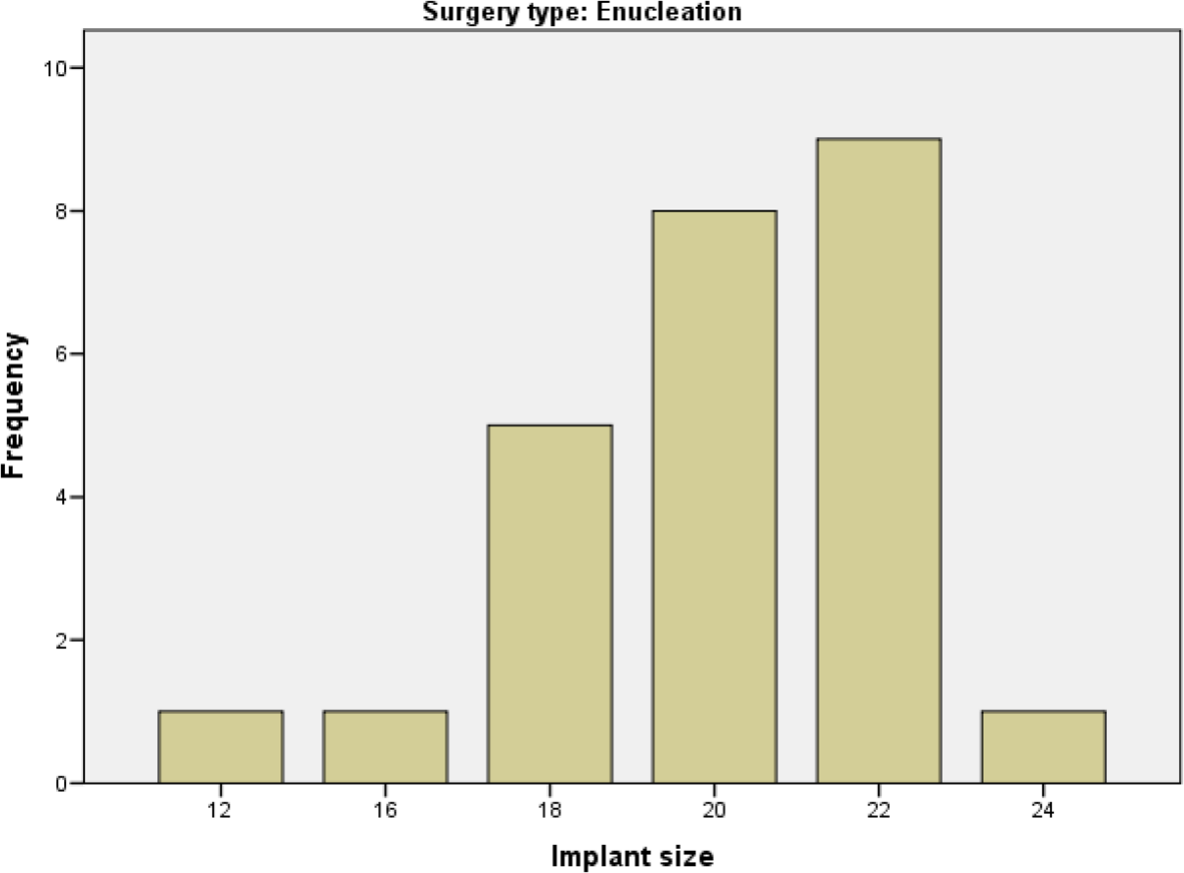
The sizes of the silicone implant spheres in millimeters and their frequency in the enucleation subgroup

All the removed eyes were replaced initially with silicone orbital implants at the time of primary surgery. The mean and median implant sizes were 16.6 and 18.0 mm respectively (range, 8–22), in the evisceration group (Fig. 1). In the enucleation group, the mean and median implant sizes were 20 mm (range, 12–24) (Fig. 2). The difference between the two groups was statistically significant (*p* = 0.001).

After anophthalmic surgery, implant exposure or extrusion developed in 8.8 % (*n* = 6/68) of cases (11.5 % [*n* = 3/26] in the enucleation group and 7.1 % [*n* = 3/42] of cases in the evisceration group). Implant migration laterally occurred in 3.8 % (*n* = 1/26) of the enucleated eyes but none in the eviscerated eyes. Ten percent (*n* = 7/68) of the entire group was complicated by partial wound dehiscence (11.5 % [*n* = 3/26] in the enucleation group and 9.5 % [*n* = 4/42] in the evisceration group) with no statistical difference found between both groups (Table 5). Overall, 67.6 % of patients (46/68) (71.4 % of the evisceration group and 61.5 % of the enucleation group) did not report any complication postoperatively during the follow-up period. Two-thirds of the complications were managed conservatively (observation for small wound dehiscence or minimal implant exposure, or with systemic antibiotics); patients with implant extrusion required replacement with a smaller implant size or a dermis-fat graft (in one patient), and 8 % required re-suturing of the wound. Sympathetic ophthalmia did not develop in any case.

## Discussion

Controversy remains regarding the advantages and disadvantages of each procedure [9, 10]. In the past, most surgeons preferred enucleation for various indications mainly because of the fear of sympathetic ophthalmia that can occur after evisceration. [11] sympathetic ophthalmia, a potentially devastating and blinding autoimmune condition characterized by panuveitis, in which the injured eye incites inflammation in the fellow sympathizing eye, was first reported in association with evisceration in 1887 [12, 13]. Even though evisceration and enucleation surgeries successfully control pain, [14] many surgeons believe that enucleation controls pain better than evisceration and evisceration is more painful postoperatively than enucleation, [14, 15] and because of the fear of sympathetic ophthalmia, [11] enucleation previously was preferred by most surgeons for various indications. However, Shah-Desai et al. [16] found that ultimate pain relief was achieved in all patients after enucleation or evisceration at an average of 3 months with no difference in postoperative pain between the eviscerated or enucleated groups [16] and recent studies have reported that evisceration is safe and associated with a low risk of sympathetic ophthalmia [17, 18].

Evisceration recently has become increasingly popular for many reasons [19]; there is no solid evidence that evisceration is associated with an increased risk of sympathetic ophthalmia [20] and the surgery requires less manipulation and consequently less inflammation and scarring of orbital tissues and extraocular muscles resulting in better implant motility and cosmetic outcome than enucleation [9, 21]. Furthermore, evisceration is simpler, faster, and associated with lower risk of bleeding intraoperatively and fewer postoperative complications, such as ptosis, implant migration, implant extrusion, socket contracture, and the deep superior sulcus syndrome [8, 21–23]. Similarly, we preferred performing evisceration rather than enucleation unless contraindicated or not feasible; therefore, 62 % of our patients in the current study underwent evisceration rather than enucleation. The most common cause of anophthalmic surgery in our series was trauma in 40 % of cases followed by a blind painful eye secondary to absolute glaucoma with phthisis bulbi and degeneration in the enucleation group and endophthalmitis in the evisceration group.

Moshfeghi et al. conducted a review of enucleation and reported trauma as the leading indication for enucleation worldwide and for 40.9 % of cases in the United States, [24] and trauma has been the leading cause for both types of anophthalmic surgeries in some reports [10, 25–29]. Similarly, trauma was the reason for most cases of anophthalmic surgery in the current series, mainly for ocular trauma patients who presented with no light perception (NLP) vision, expulsion of the intraocular contents, and lacerations involving zone III. Trauma accounted for 40 % of the indications in our series (50 % in the enucleation group, and 33.3 % in the evisceration group). In cases with extensive globe disruption, removal of all uveal tissue may be difficult via evisceration; therefore, enucleation may better safeguard against retained uveal tissue. However, in cases in which the sclera is largely intact, and the intraocular contents are contained and identifiable, evisceration may be a reasonable alternative based on surgeon preference and experience [30, 31]. However, post-traumatic early enucleation or evisceration should not be performed because of an initial vision of NLP alone, since Agrawal et al. [32] found that one third of traumatized eyes with a preoperative visual acuity of NLP had ambulatory vision or better after surgery. Other reports also showed improvement of NLP vision after surgical repair due to advances in vitreoretinal surgery [33–35]. Moshfeghi et al. [24] also reported that intraocular tumors were the second leading cause of enucleation in 24 to 28 % of cases, while only three patients in the current series had tumors (two retinoblastoma and one choroidal melanoma). Intraocular tumors accounted for 4.4 % of anophthalmic surgery in the current series since almost all cases of ocular tumors were referred and managed in a nearby, specialized cancer center in Jordan that was established in 1997 [36].

The complication rates of enucleation and evisceration have ranged from 6 to 100 %, with erosion being the most commonly encountered complication [24, 29, 37]. In the current series, around 1/3 of patients reported minor or major local side effects, with wound dehiscence and implant exposure being the most common (Table 5). Even though insertion of the largest implant possible, whether during an enucleation or evisceration procedure, may be associated with increased risk of implant exposure or extrusion, it can prevent enophthalmos and superior sulcus deformity;[3] therefore, recent advances in evisceration techniques largely focused on various types of posterior sclerotomies to allow for placement of larger implants (up to or even larger than 20 mm) in a large percentage of patients [30, 38].

The impact of suturing the muscles in relation to the implant for the sake of prosthesis motility after enucleation is still a controversy in the literature. Some surgeons are suturing the muscles directly to the implant or to a mesh around the implant, [29, 39, 40] while others suture the muscles together in front of the implant (imbrication) [41]. In the other hand, some surgeons prefer the myoconjunctival technique in which they suture the muscles to the conjunctiva in the fornix rather than to the implant and they report motility that is better than suturing the muscles in front the implant and equal motility (with less migration, and exposure) to direct suturing of the muscle to an integrated implant [42, 43]. In another technique (as we did in this series) the muscles were cut near their insertions to the globe and left without suturing to the implant nor in front of the implant, with meticulous suturing of the tissues anterior to the implant to decrease the risk of implant extrusion or exposure. No single eye in this series had contracted socket, and this is not unexpected for us basically since no single patient in this series had received radiation which is the most important risk factor for contracted socket.

In cases of endophthalmitis, evisceration is preferred to enucleation because evisceration is thought to have less risk of postoperative meningitis or encephalitis [44]. In the current series,12 of the 13 patients with endophthalmitis (92 %) underwent evisceration with silicone sphere orbital implant insertion at the time of primary surgery in an attempt to save the patient a secondary implant insertion, which recently has been reported to be most successful [4, 23, 44, 45]. We also did not notice an increased risk of infection or implant extrusion in these cases. Postoperative orbital infection developed in three cases in the current series, i.e., in one patient after a massive globe injury during a motor vehicle accident with eyelid swelling and orbital pain 3 months after enucleation and in one patient each after keratitis and endophthalmitis. All three cases were treated conservatively with oral antibiotics without removal of the orbital implant.

In the current series, the enucleation group was significantly younger than the evisceration group (25.24 ± 16.50 years compared to 47.12 ± 24.11 years, *p* = 0.001), which may be due to trauma and tumors; 23 of the trauma patients were younger than 50 years and only four patients were older than 50 years (Table 3), and due to enucleations for retinoblastoma being done at younger ages. Furthermore, patients who developed endophthalmitis and keratitis usually had previous intraocular surgery and are of older age that primarily underwent evisceration. In addition, since about two-thirds of our trauma cases where men, we had a male preponderance with a ratio of about 2:1, similar to other reports [31, 46, 47].

## Conclusion

Our results were similar to other reports from developed and developing countries. The major causes for anophthalmic surgery remain trauma, a blind painful eye, and endophthalmitis. Patients who require enucleation (mainly as the result of trauma and tumors) were younger and had larger implants than the patients who underwent evisceration.
